# Hierarchical integration of porosity in shales

**DOI:** 10.1038/s41598-018-30153-x

**Published:** 2018-08-03

**Authors:** Lin Ma, Thomas Slater, Patrick J. Dowey, Sheng Yue, Ernest H. Rutter, Kevin G. Taylor, Peter D. Lee

**Affiliations:** 1 School of Earth and Environmental Sciences, The University of Manchester, Manchester, M13 9PL UK; 2 Manchester X-ray Imaging Facility, School of Materials, The University of Manchester, Manchester, M13 9PL UK; 3 Research Complex at Harwell, Harwell Campus, Oxfordshire, OX11 0FA UK; 4 Department of Mechanical Engineering, University College London, London, WC1E 7JE UK

## Abstract

Pore characterization in shales is challenging owing to the wide range of pore sizes and types present. Haynesville-Bossier shale (USA) was sampled as a typical clay-bearing siliceous, organic-rich, gas-mature shale and characterized over pore diameters ranging 2 nm to 3000 nm. Three advanced imaging techniques were utilized correlatively, including the application of Xe^+^ plasma focused ion beam scanning electron microscopy (plasma FIB or PFIB), complemented by the Ga^+^ FIB method which is now frequently used to characterise porosity and organic/inorganic phases, together with transmission electron microscope tomography of the nano-scale pores (voxel size 0.6 nm; resolution 1–2 nm). The three pore-size scales each contribute differently to the pore network. Those <10 nm (greatest number), 10 nm to 100 nm (best-connected hence controls transport properties), and >100 nm (greatest total volume hence determines fluid storativity). Four distinct pore types were found: intra-organic, organic-mineral interface, inter-mineral and intra-mineral pores were recognized, with characteristic geometries. The whole pore network comprises a globally-connected system between phyllosilicate mineral grains (diameter: 6–50 nm), and locally-clustered connected pores within porous organic matter (diameter: 200–800 nm). Integrated predictions of pore geometry, connectivity, and roles in controlling petrophysical properties were verified through experimental permeability measurements.

## Introduction

Shales constitute two-thirds of the volume of sedimentary rocks yet are arguably the least well understood rock type of the sedimentary record^1^. Additionally, these rocks are of particular importance as an economic resource, but exploitation may pose certain environmental hazards^2–4^.

Shale can be a source rock for conventional hydrocarbon systems, a storage reservoir rock for self-sourced hydrocarbons^4,5^, a target for geo-thermal energy^6^, and also cap rock for hydrocarbon reservoirs, carbon sequestration and nuclear waste disposal^3,7^. An accurately quantified pore model is important for understanding and enhancing the above applications. The key properties of shales that underpin commercial viability and minimize environmental impact, such as permeability, storativity, elastic properties and electrical conductivity, are directly related to pore-size distribution, pore geometry and the connectivity of the pore network^8,9^. For example, the location of nano-pores associated with organic matter or phyllosilicate minerals are pivotal not only for understanding pore generation and evolution during maturation and fracturing^10^, but also in controlling and influencing gas transport and storage^11^.

Nevertheless, there remains a poor understanding of the full characteristics of pore systems in shales. Systematic quantification of pore geometry and detailed imaging of pore networks in shales, relative to the locations of pores, are rare. Three dimensional (3D) imaging of pores has been reported for a number of different shales^12,13^, but no integrated pore models have been built to cover the wide range of pore size. A common characteristic of prior studies has been to build a micro-scale dataset, combined with relatively low-resolution nano-scale datasets using X-ray tomography (XCT) and Focused Ion Beam - Scanning Electron Microscopy (FIB-SEM)^14–16^. An important consensus has emerged that pores greater than tens of nanometres in size do not display a connected pore network. Whether a connected pore network is formed by pores below 10 nm, however, has not been confirmed through imaging studies due to resolution limitations. Existence of nano-scale pores can be detected or inferred from some laboratory experiments, such as nitrogen adsorption, mercury intrusion porosimetry (MIP) and small- to ultra-small-angle neutron scattering (SANS–USANS)^17^, but they have not been directly imaged in 3D. Direct measurements of permeability and acoustic wave velocity anisotropies, and their pressure sensitivity, on bulk shale samples^11^ have also led to the inference that highly anisotropic, nano-scale pores of low aspect ratio must control transport properties, whilst the larger pore sizes control overall gas storage capacity.

Advanced multi-scale and multi-dimensional imaging of shale has provided opportunities to image pores, organic matter and minerals over a range of scales^16,18,19^. However, the geometry and connectivity of these pores were not tied to quantitative measurements, which in shale systems is very complex. Extremely high-resolution (nanometre to sub-nanometre) techniques are required to image nano-pores, whilst a large field of view (tens of microns) is needed to provide a representative imaging volume. This requires high-resolution electron microscopy (EM) such as scanning electron microscope (SEM) and transmission electron microscope (TEM) instead of X-ray tomography, as they are able to provide mineral and organic matter information which cannot be acquired using present X-ray tomography techniques, due to their lower contrast and resolution^20^. Consequently, there is a need for a combination of electron microscopy techniques that span a wide range of scales to visualize the geometry and networking of all pore types.

Traditional planar TEM results have allowed identification of individual nano-pores^21,22^, but did not document spatial geometry and network connectivity. Advanced TEM tomography can provide down to 2 nm resolution (sub-nanometre voxel sizes) within a sample a couple of hundred nanometres thick. The advanced Xe^+^ Plasma FIB (PFIB) permits mass removal rates at least 60× greater than conventional Ga^+^ FIB systems with comparable or less damage^23^. It makes it possible to combine a large field of view (tens to hundreds of microns) with high-resolution SEM images (tens of nanometres) at the same site. This can be further combined with conventional Ga^+^ FIB systems, to allow specific sites to be imaged with a medium field of view (a few microns) and high resolution SEM (a few nanometres) images.

Using these techniques, this study aims to build an integrated geometric and network model of a representative shale sample based on pore occurrence using a principal component analysis (PCA) method to reconstruct the pore system, and to construct models incorporating properties of size, geometry and network connectivity. The sample was selected with typical TOC, maturity, composition and micro-texture from the Haynesville-Bossier Shale (a major US shale gas play). We explore the potential of Scanning (S)TEM tomography to provide nanometre resolution images for nano-pores and present the first-time application of Xe^+^ Plasma FIB (PFIB) slice-and-view imaging in shale to provide volumes with a large field of view, further combining these with Ga^+^ FIB slice-and-view imaging to bridge the associated length scales. These novel imaging and correlative techniques provide pore images over a large size range, from ca. 2 nm to 2 microns, covering the great majority of pores in shale over a large volume. Also, four different types of pores and their three distinct size categories were characterized using an improved principal component analysis (PCA) method, allowing pore models to be constructed that incorporate variability in size, geometry and network connectivity. The results will not only lead to an improved understanding of the pore network in shales across a wide range of scales, but also provide the fundamental parameters for modelling work.

## Results

### 3D pore visualization across scales

**Figure 1:**
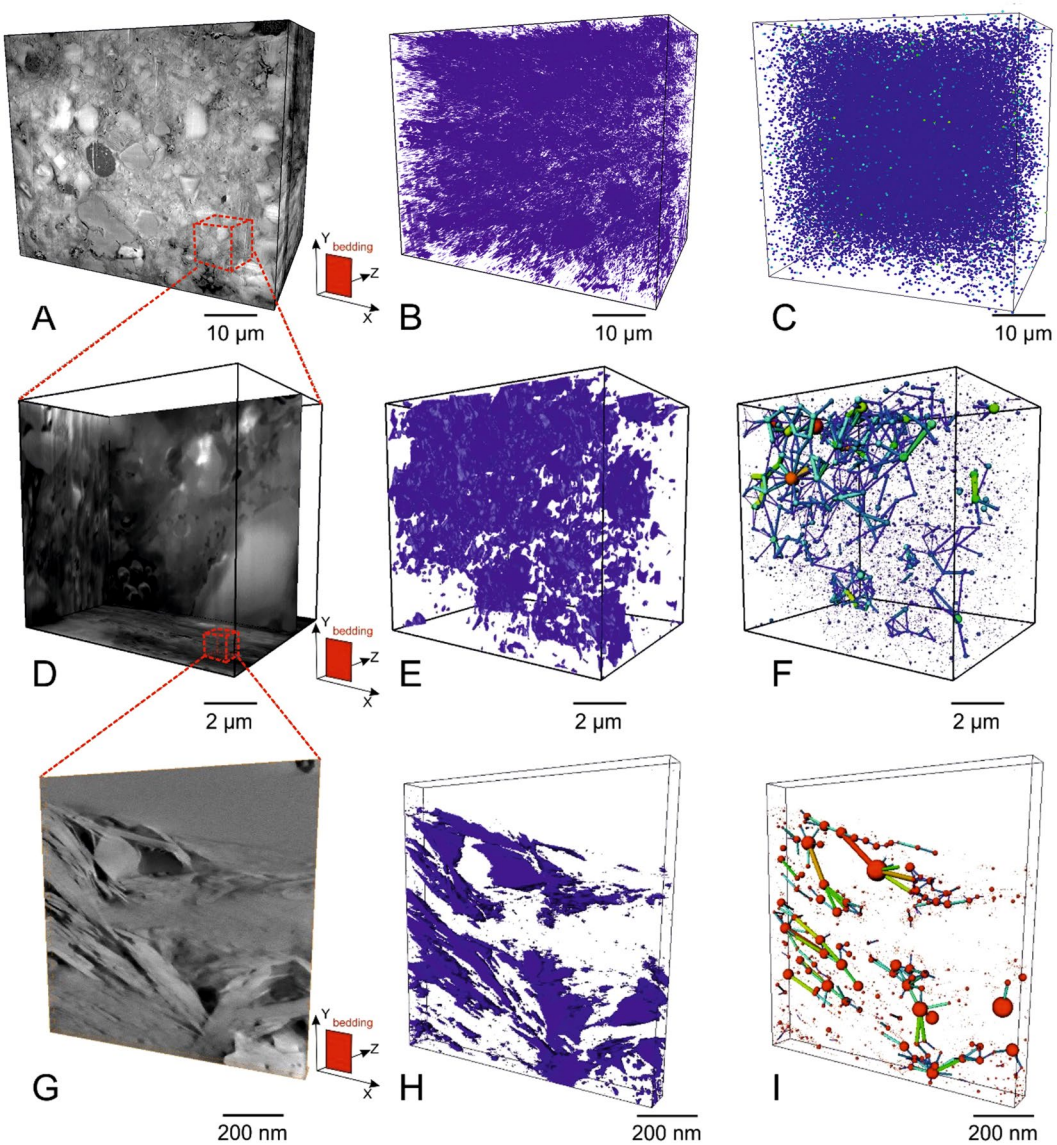
3D SEM and STEM images of pores and pore networks with varying degrees of connectivity (perspective projections). The front-left plane of these images is parallel to bedding and the scale bars apply equally in depth as well as to frontal area. (**A**–**C**) are from P-FIB images, with physical sizes 80 × 65 × 40 µm^3^ and voxel sizes 16 × 13 × 50 nm^3^, (**D**,**E**,**F**) are from FIB images, with physical sizes 10 × 10 × 5 µm^3^ and voxel sizes 10 × 10 × 20 nm^3^. (**G**–**I**) are from STEM tomography images, with physical sizes 0.6 × 0.6 × 0.06 µm^3^ and voxel sizes 0.6 × 0.6 × 0.6 nm^3^. (**A**,**D**,**G**) are orthogonal slices of the 3D images, (**B**,**E**,**H**) are segmented pores in 3D, (**C**,**F**,**I**) are representations of the connectivity of the extracted pore network in 3D view, with the relative sizes of the pore volumes represented as equivalent spheres. The small dashed line boxes (**A**,**D**) demonstrate the relative sizes of the higher resolution technique, rather than the specific relative locations of the images. Bedding plane orientations are shown in (**A**,**D**,**G**).

Pores were imaged at three scales, with PFIB images, FIB images and STEM images, with spherical-equivalent voxel diameters 22 nm, 13 nm and 0.6 nm, and physical volumes 20000 µm^3^, 500 μm^3^, and 0.02 μm^3^, respectively (Fig. 1). The front-left plane of these images is parallel to bedding and the scale bars apply equally in all dimensions.

To identify individual pores and their connectivity, the pore volumes were separated to individual pores and throats. The number of connecting pore throats were counted for each pore and this becomes the ‘coordination number’ for that pore. The ‘size’ of an individual pore was determined as the diameter of a sphere (equivalent diameter) containing the same number of voxels. The ‘shape’ of an individual pore was characterized using Principal Component Analysis^24^ in which each pore is represented by the ratio of orthogonal principal axes *a*, *b* and *c* of an ellipsoid representing the departure from sphericity (see Supplementary materials Figure S3) and its orientation. The longest axis is *a* and the shortest is *c*, and the intermediate *b* axis is orthogonal to the *a/c* plane.

Pores greater than 66 nm equivalent diameter were quantified in 3D in the PFIB images after noise removal. PFIB-imaged porosity was measured to be 4.6%, with the largest pore size being 1756 nm in diameter, but no continuous pore network was found in the mineral matrix at this scale (Fig. 1A–C).

In FIB images, pores were observed to be locally connected in the porous organic matter, and some of them are connected to other pores in the matrix (Fig. 1D–F). The majority of pores in the phyllosilicate matrix are isolated at this scale. Connected pore networks were found in the phyllosilicate mineral matrix and inside single organic matter particles in (S)TEM images, with voxel sizes (spherical-equivalent voxel after noise removal) down to 0.9 nm (Fig. 1G–I).

This voxel size is close to the size of one methane molecule 0.4 nm^25^, although the true resolution in the 3D reconstruction is closer to 2 nm (Figure S2 in supplementary materials). Nevertheless, the pore network extracted from these images should be reliable for gas transport modelling.

### Pore size distributions

**Figure 2:**
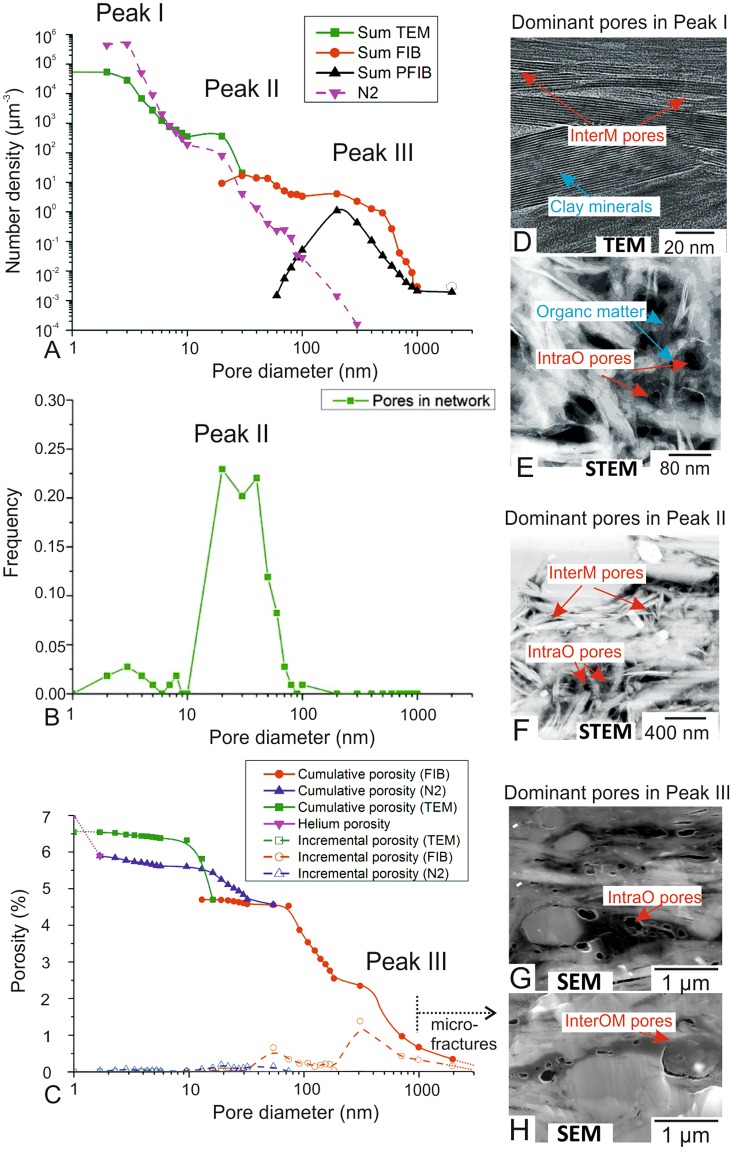
Pore size distributions and corresponding pore types from 1 nm to 3000 nm. (**A**) Number density extracted from 3D images and nitrogen adsorption, (**B**). Frequency in connected network (**C**). Cumulative volume fractions of 3D images, nitrogen adsorption and helium porosity, (**D**–**H**) shows the dominant pore types corresponding to three peaks.

Pore sizes were measured using 3D image analysis, nitrogen adsorption and helium porosity. Those from images are displayed by number density (number of pores/µm^3^) to provide the same standard for the three scales (~1–3000 nm; Fig. 2A,C). They are compared with pore sizes quantified by the Barrett–Joyner–Halenda (BJH)^26^ nitrogen adsorption method in the range of 2–300 nm (Figure A and C). The peaks identified using these techniques show similar trends (Fig. 2A) in the overlap ranges (2–300nm). The pores of sizes larger than 300 nm are shown by PFIB and FIB data and those below 2 nm are supplemented by helium porosity data (Fig. 2A,C). Nitrogen adsorption covers a significant range of sizes, but cannot provide information on pore location, geometry, orientation, network and other details which can be measured utilising 3D images acquired in this study. Fractures, quantified to be a few microns in aperture^27^, were ignored as they are normally considered as arising during depressurisation, and assumed to be closed under subsurface pressure conditions^11,28^.

The major peak of pore size number density appears around 2–3 nm (Peak I in Fig. 2A), and the second peak is around 20 nm (Peak II in Fig. 2A,B). A third peak is observed at 100 nm (Peak III in Fig. 2A,C). These match each of the three image datasets and the first two peaks can also be found in the nitrogen adsorption data. Peak I can be only acquired from TEM images, including pores between phyllosilicate mineral grains or within some organic matter particles (described as InterM and IntraO respectively in the next section) (Fig. 2D,E). Peak II is seen primarily in the FIB dataset, corresponding to a platform in TEM dataset. Most of these pores occur between mineral particles (described as InterM in the next section) (Fig. 2E). Peak III is consistent in the FIB and PFIB data. Pores inside organic matter particles or between organic matter and minerals (described as IntraO and InterOM in the next section) (Fig. 2G,H) contribute a large portion of this peak.

**Figure 3:**
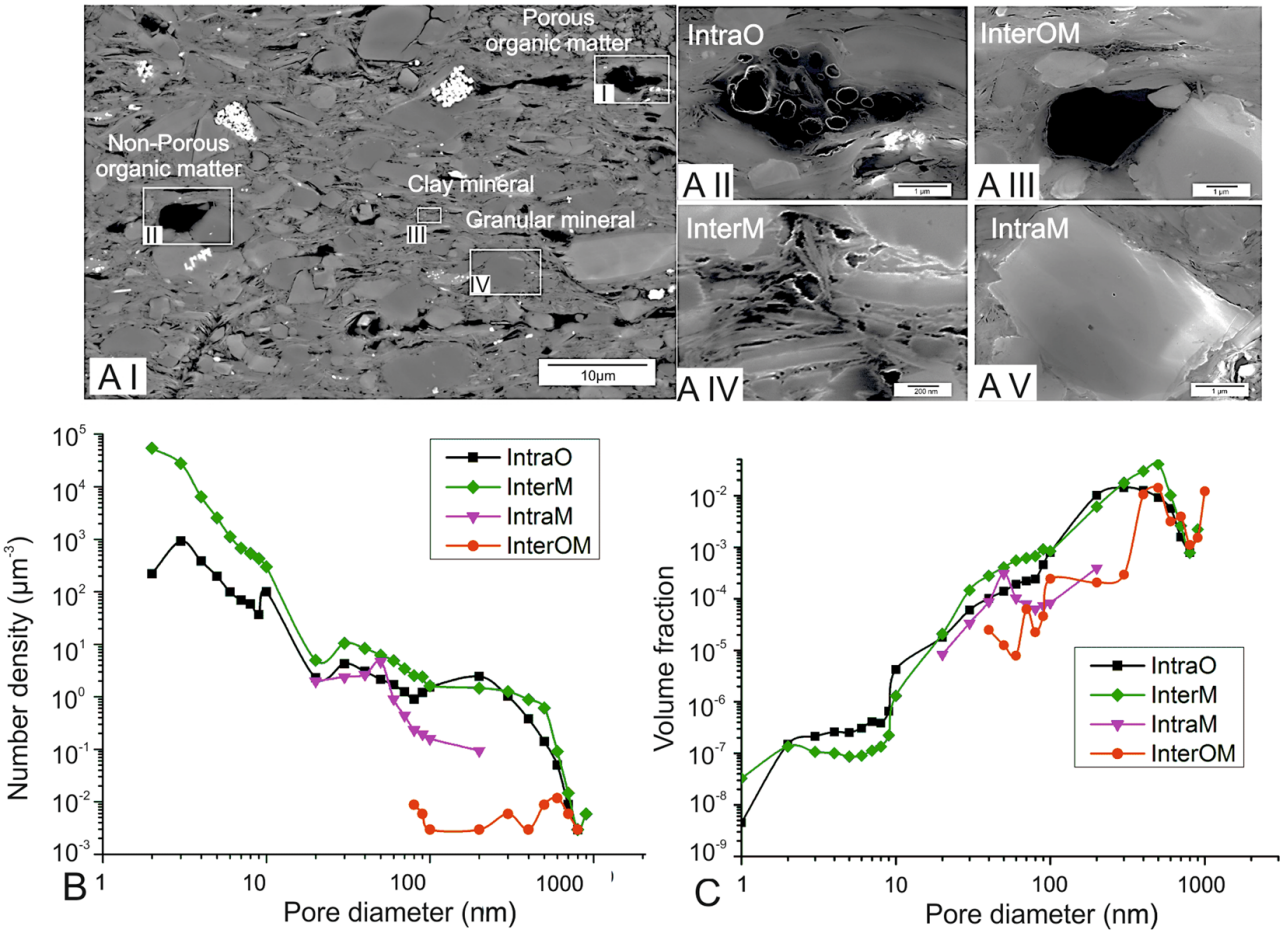
SEM observation, pore size distributions, volume fractions of four pore types. (**A**) Porosity-associated phases and corresponding pore types. (**B**,**C**) Pore size distribution displayed respectively by number density volume fraction.

Pores with coordination numbers greater than 2 are considered to form part of a connected network. Their distribution shows a peak at around 10–100 nm (Fig. 2B), corresponding to the Peak II in Fig. 3A. 87% of pores with coordination number >2 appear in the range 20–60 nm.

The pore size occurrences by frequency were converted into volume fractions (Fig. 2C). Pores >100 nm equivalent diameter make the greatest contribution, accounting for 57% of total helium porosity, and 3.8% of total rock volume. Pores of 10 to 100 nm occupy 39% of the total porosity, and 2.6% of the total rock volume. Pores below 10 nm, only occupy 3% of the total porosity, and 0.2% of total rock volume.

In summary, Peak I pores contribute the greatest number (Fig. 2A), Peak II pores contribute most towards the connected pore network (Fig. 2B), while Peak III pores contribute the most in volume (Fig. 2C).

### Porosity-associated phases and corresponding pore types

Pores are associated with organic matter, mineral grains, and the phyllosilicate mineral-dominated matrix, which we define as ‘porosity-associated phases’ (Fig. 3A). Two typical types of organic matter were identified: porous organic matter and non-porous organic matter (Fig. 3AI,II). Porous organic matter particles contain large, connected pores (Fig. 3AI), whereas non-porous organic matter has crack-like pores aligned along the edge of the phase, but contains no resolvable internal pores (Fig. 3AII). Phyllosilicate minerals include illite, chlorite and muscovite (Fig. 3AIII), and granular minerals are defined here to include quartz, calcite, ankerite, albite and pyrite (Fig. 3AIV). Imaging results show that pore geometry and network properties vary according to the phases associated with each type of pore. Based on the relationship of pores and associated phases, pores are classified into four types: intra-organic matter pores (IntraO), organic-mineral interface pores (InterOM), inter-mineral pores (InterM) and intra-mineral pores (IntraM) (Fig. 3AI–IV; for more images see Figures S1-S2 in supplementary materials).

#### Porous organic matter and Intra organic matter pores (IntraO)

IntraO pores are defined as pores completely bounded by organic matter. ‘Porous organic matter’ refers to organic matter particles containing at least one IntraO pore. A number of organic matter particles are randomly-arranged in the matrix (Fig. 3AI and AII; more images see Figure S1 in supplementary materials). Most porous organic matter particles are lamellar in shape, and some are interlayered with phyllosilicate minerals. IntraO pores are locally connected within the organic particle, and appear in clusters with pyrite framboid-like forms. Pore sizes are relatively uniform in each particle.

#### Non-porous organic matter/mineral interface pores (InterOM)

Organic- mineral interface pores (InterOM) are defined as pores at the interface of organic matter with mineral particles. Non-porous organic matter is dominated by spheroidal or irregular polygonal particle shapes (Fig. 3AI and AIII; more images see Figure S1 in supplementary materials). However, pores <2 nm are unlikely to be resolved in the disordered organic matter. InterOM pores take typically curved flake-like forms or possess a crack-like geometry, aligned along the edges of non-porous organic matter particles, commonly parallel to the trace of bedding.

#### Phyllosilicate minerals and inter-mineral pores (InterM)

Phyllosilicate mineral grains, dominantly illite, chlorite and muscovite, are oriented subparallel to the bedding plane (Fig. 3AI and AIV; more images see Figure S2 in supplementary materials). InterM pores occur within clusters of mineral grains, either between phyllosilicate grains or between phyllosilicates and other mineral grains. Most InterM pores have elongated wedge shapes, normally slightly larger when fine-grained granular minerals (e.g. quartz) occur within the phyllosilicate mineral matrix.

#### Granular minerals and intra-mineral pores (IntraM)

Quartz, calcite, ankerite and albite were commonly found in this sample, based on the XRD data and imaged morphologies. IntraM pores are completely encased within individual minerals or clusters. They are generally small and have spheroidal or irregular polygonal shapes (Fig. 3AI and AV; more images see Figure S2 in supplementary materials). However, IntraM pores do not form interconnected pore networks. Pyrite framboids host polygonal-shaped, crystallographically-controlled pores. Single pyrite crystals do not host pores. Pores in pyrite framboids can be interconnected, but do not contribute significantly to porosity as pyrite occurs only in trace concentrations (<1 wt%).

Only IntraO and InterM pores >20 nm are seen in TEM images. Pores with a 2–3 nm diameter have the largest number density (µm^−3^) for these types (Fig. 3B). A second concentration occurs at 30 nm. IntraO pores have a third maximum around 200–300 nm while the InterM curve flattens after the second concentration maximum and then decreases rapidly at sizes >500 nm. IntraM pores are relatively few compared to InterOM, only appearing in significant number densities at 20–200 nm with a peak around 50 nm. InterOM pores are observed only in small concentrations for diameters of 80–2000 nm. Volume fractions of IntraO, InterM and InterOM pores are greatest at 300–900 nm, while the IntraM pore sizes peak at 50 nm (Fig. 3C).

### Pore geometry model

**Figure 4:**
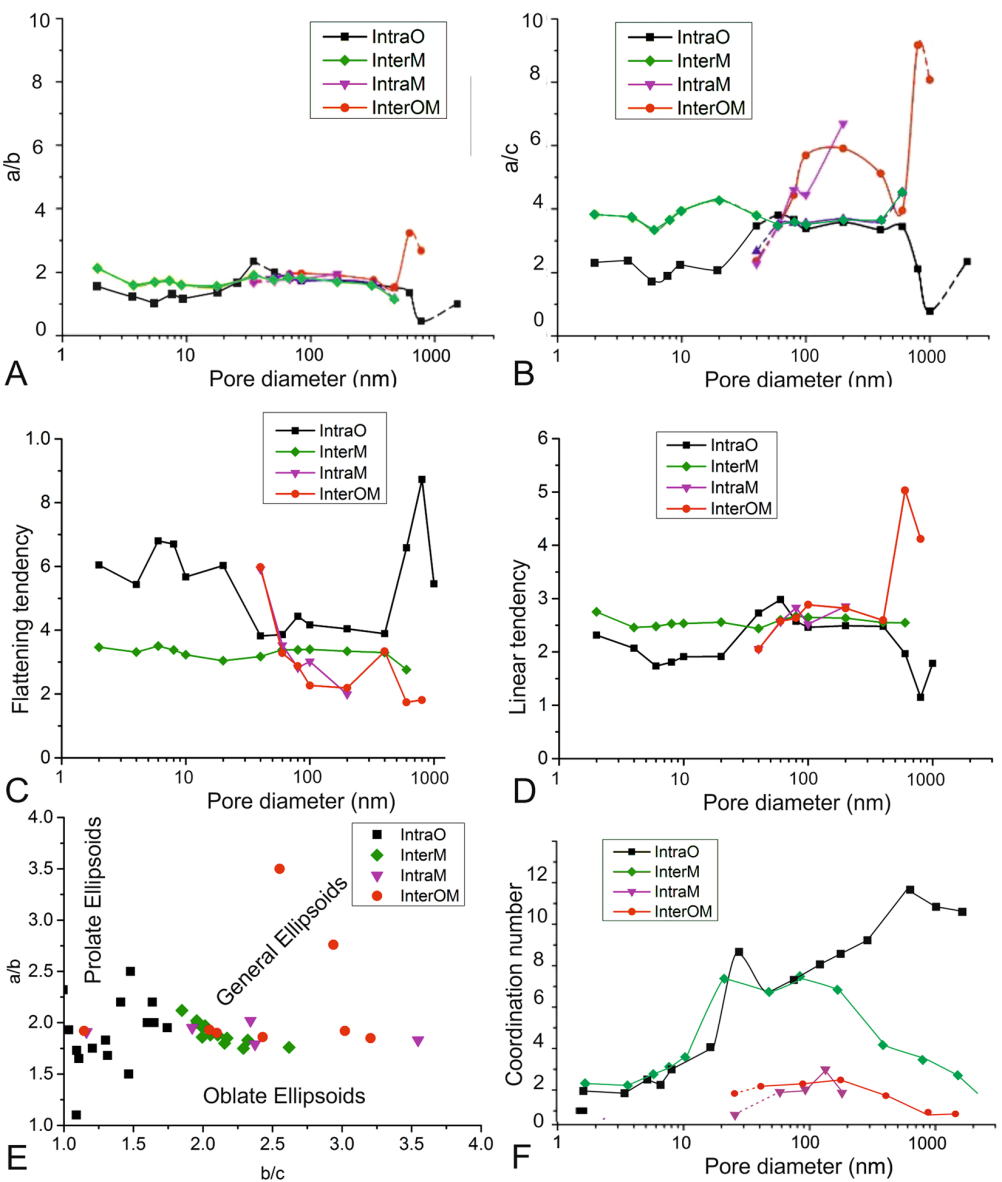
Pore shape distributions for the four identified pore types displayed averaged according to volume fraction classes. Each class contains hundreds to thousands of pores. (**A**,**B**) Show PCA axial ratios a/b and a/c. (**C**,**D**) Show respectively the tendency for pores to be flattened (values < 1) and elongated (values > 1). (**E**) Is a Flinn plot; pores close to the vertical axis tend to be prolate ellipsoids; those along the horizontal axis are oblate elipsoids, and general ellipsoids lie in the intervening space. Shape eccentricity increases with radial distance from (1, 1). (**F**) shows coordination number (number of connections to adjacent pores) as a function of pore size.

The shapes of individual pores in each pore type category from Principal Component Analysis can be conveniently described by plotting axial ratios, for example *a/b* and *a/c* (Fig. 4A,B). The data for each pore type were averaged for each of a series of size categories on a logarithmic scale to base 2.

The 3-dimensional shapes of ellipsoids representing the whole spectrum of pore shapes can be represented by plotting *a/b* versus *b/c*^29^ (Fig. 4E). The origin (1, 1) represents spheroidal pores; points along the vertical axis represent uniaxial prolate ellipsoids and along the horizontal axis uniaxial oblate ellipsoids. The intervening space represents general triaxial ellipsoids, and radial distance from the origin corresponds to increasing eccentricity (increasing flattening or stretching). IntraO pores are near–spheroidal with a tendency to prolateness. InterM pores are near spheroidal, triaxial ellipsoids. IntraM and IntraOM pores show a tendency towards more eccentric and flattened shapes, especially for the intermediate and larger pore sizes, which also tend to be the best connected (larger coordination numbers, Fig. 4F).

The tendency towards flattening (*S*) or elongation (*L*) of individual pore shapes can be represented in terms of axial ratios using the functions

$$S=2/(a/c+b/c)\,and\,L=2/(b/a+c/a)$$

where *S* <1 for more flattened shapes, and *L* >1 for more elongate shapes. These functions are plotted versus log grain size in Fig. 4C,D respectively. Substantial deviations from near constant values occur in both cases only for large pore sizes. IntraM pores in both cases show the smallest deviations from sphericity, although they are still triaxial ellipsoids. The other three pore types exhibit comparable triaxial shapes, with both flattened and elongated forms. InterOM pores exhibit the greatest degrees of both flattening and lineation development, but only at the largest pore sizes (>100 nm).

Figure 4D shows the variation of coordination number for the four pore types versus grain size. IntraO and InterM pores are best connected (Coordination number ~1) at ca 20 to 200 nm pore size but IntraO pores are best connected at ca 1000 nm pore size, but only within the organic particles. Only the InterM pores can form extensive connected networks throughout the whole rock, and hence control the permeability. The orientations of pores are best appreciated from the SEM imagery (e.g. Fig. 3AI) and tend to be flattened in the plane of bedding, reflecting the flattening strain induced during the diagenetic compaction of the rock.

**Figure 5:**
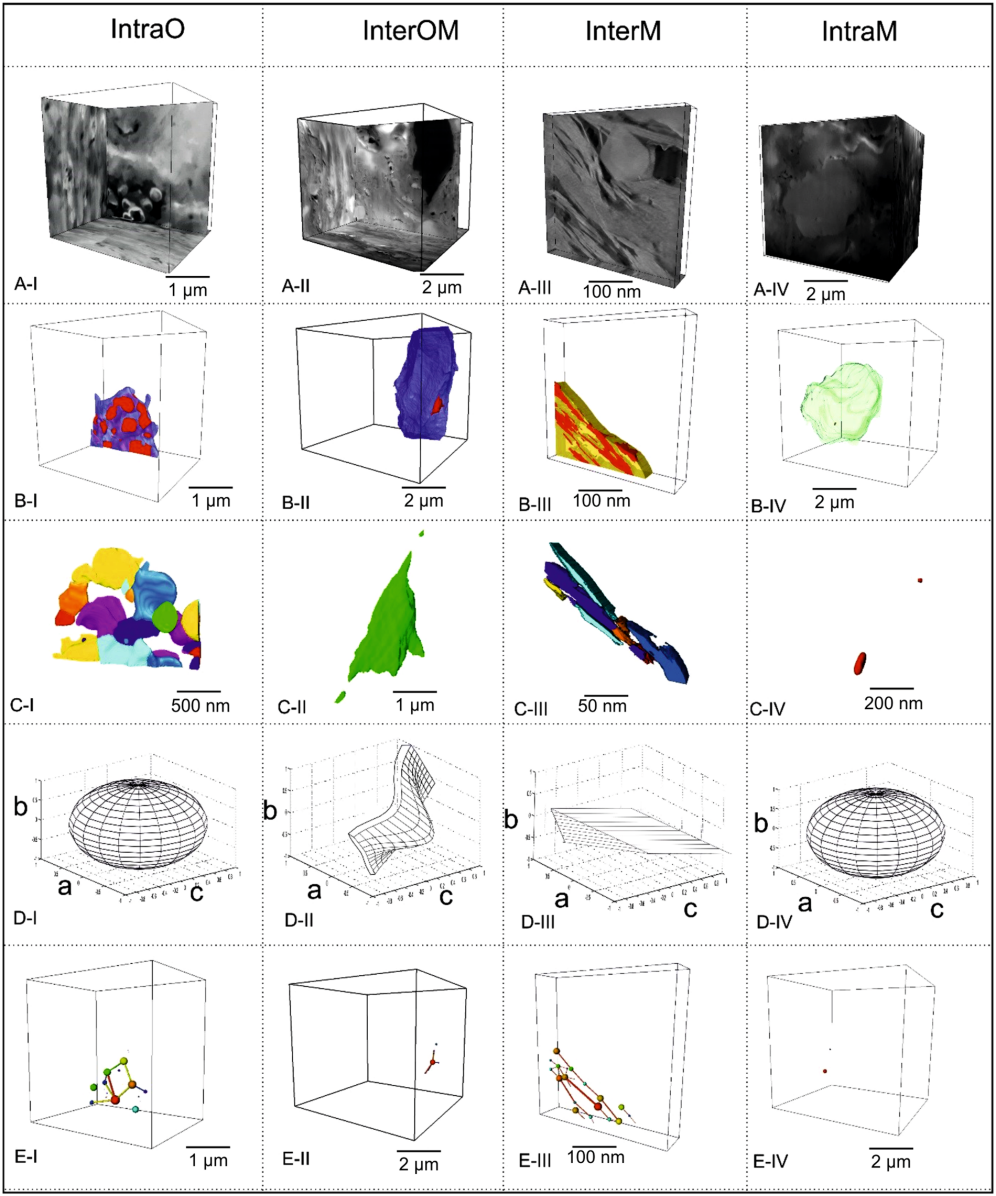
Pore geometry and coordination number models based on 3D SEM and 3D TEM images of four pore types (all are perspective projections of varying depths). In each case the plane of the bedding is sub-parallel to the front, left face of the box. For clarity, only a proportion of the pores in each box are shown. (**A**) SEM/TEM slices, (**B**) segmented organic matter/mineral grains in relation to neighbouring pore spaces: red- pore spaces, blue- organic matter, yellow- phyllosilicate minerals, green- granular minerals. (**C**) separated pores to illustrate geometry, separate colours refer to individual pores, (**D**) idealized shapes representing the four geometric type models, with reference to principal axes *a*, *b* and *c*. (**E**) Illustration of coordination number concept for the pore/grain relations illustrated in B; balls mark centroids of pores of various sizes, lines show connections between pores. Only pore type InterM shows development of a widespread, spatially-connected network.

Each of the four pore types contributes a proportion to the overall rock volume, pore number density and to characteristic shapes and network connectivity (Fig. 5), although the descriptions of pore networks are not very reliable for pores at the two ends of the scale for each image, due to the limitations of total volume and voxel sizes. These are described below and summarized in Table 1.

**Table 1:** Summary of pore characteristics and measurements.

Pore types		IntraO	InterOM	InterM	IntraM
Associated phases		porous organic matter	non-porous organic matter	Phyllosilicate minerals	Granular minerals
Fractions	Frequency	2.2%	97.7%	<0.1%	<0.1%
	Volume fraction	25.8%	51.9%	0.6%	21.7%
	Porosity fraction	1.8%	3.6%	0.1%	1.5%
Geometric parameters		Ellipsoid	Lamellar	Elongated wedge	Ellipsoid
	a/b	1.38	2.63	1.17	1.61
	a/c	2.78	11.11	4.17	3.13
	Orientation	// bedding	vary	// bedding	vary
Network parameters^*^	Number fraction	13.6%	1.2%	14.2%	4.5%
	Volume fraction	80.2%	2.8%	97.9%	24.6%
	Average coordination number	3.1	2.07	3.6	2.5

^*^Only for connected pores (coordination number >2). The fractions are the percentages of pores of stated type.

InterM pores display wedge shapes elongate in the trace of bedding. They comprise 97.7% of total pore number but only 51.9% of total porosity, occupy 3.6% of the total sample volume and form a bedding-parallel, web-like connected network at tens to hundreds of nanometres scale across the sample (Fig. 5).

IntraO pores only account for 2.2% of the total frequency of number of pores, 25.8% of the total porosity, and 1.8% of the whole sample volume. They tend to be larger than the other three pore types; the largest observed equivalent diameter is 1796 nm. 13.6% of IntraO pores have a coordination number greater than 2, and an estimated 80.2% of their total volume is locally connected (Table 1) to form a clustered arrangement within organic matter particles of the larger sizes, that is up to 1000 nm. These clustered pores are connected to the InterM web but are not themselves globally connected.

InterOM pores are frequently present as curved or crack-like lamellae with the *c* dimension tending to lie normal to bedding. They have a low number frequency of less than 0.1% and occupy 0.6% of the total porosity and 0.1% of the whole sample volume. Only 1.2% of InterOM pores have coordination numbers greater than 2, and they occupy 2.8% of the total pore volume (Table 1).

IntraM pores have typically polygonal geometries, and the lowest frequency fraction (<0.1%). The volume fraction of the total porosity is 21.7%, and they comprise only 1.5% of the total rock volume. IntraM pores are small and display a narrow size distribution range, 20 nm to 200 nm. Coordination numbers are 0–2 and the pores are largely isolated. Only 4.5% of these pores (by number) have coordination numbers greater than 2 and they occupy 24.6% in volume of the connected pore network (Table 1).

### Pore network reconstruction across scales

**Figure 6:**
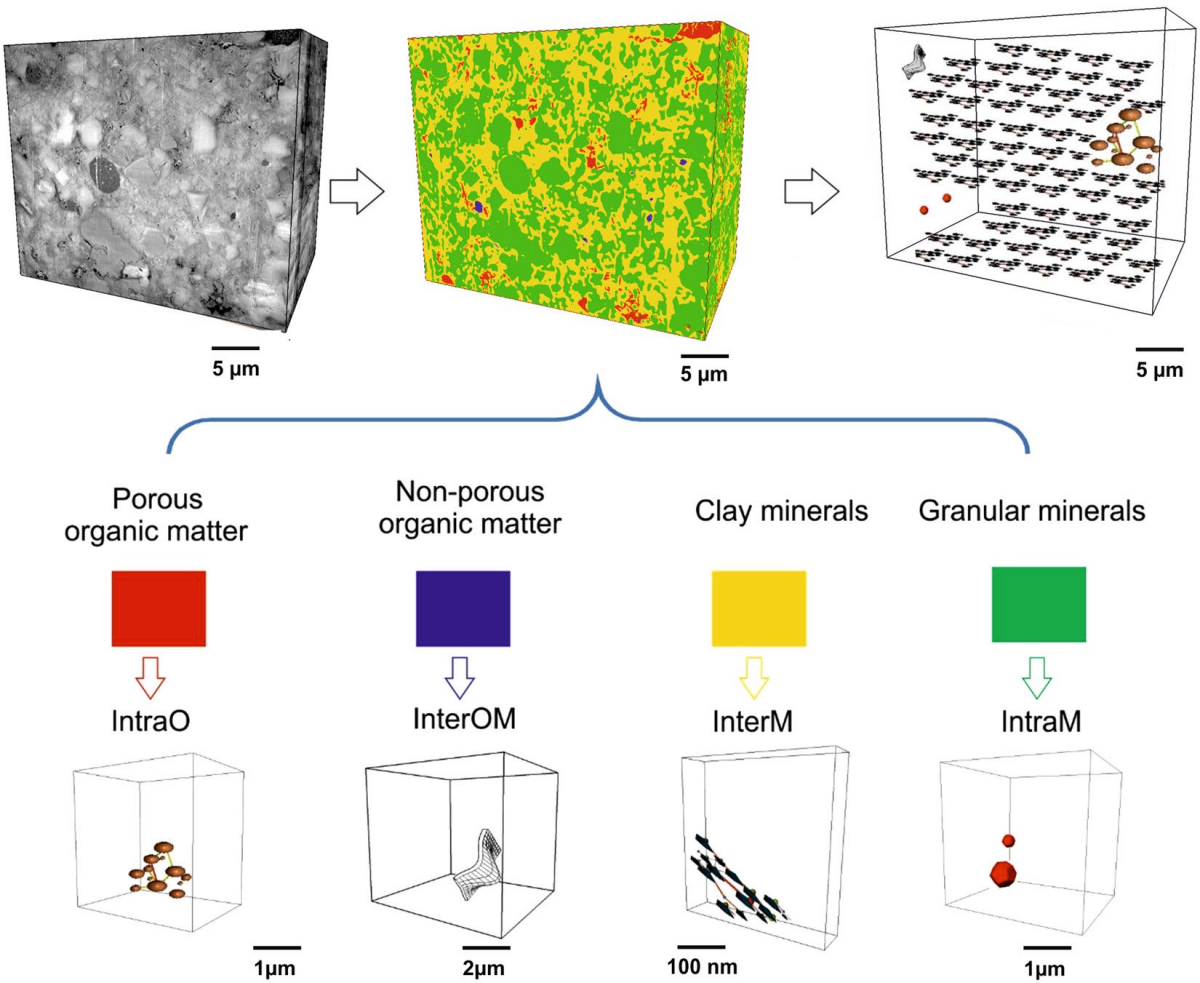
Schematic diagram of reconstructed pore networks based on the imaged pore models (perspective projections). Four phases hosting pores were identified in the lower resolution images (i.e. PFIB): porous organic matter, non-porous organic matter, phyllosilicate minerals and granular minerals. Geometry and network models of four corresponding pores were extracted in the higher resolution images (i.e. FIB and TEM images), and then upscaled into images with length scale tens of microns (i.e. PFIB).

Using these observations linked across scales, an integrated pore network can be constructed based on the geometric models and network model for the four pore types. The four pore associated phases: porous organic matter, non-porous organic matter, phyllosilicate minerals and granular minerals, are segmented from the large scale images (i.e. PFIB image). Quantified parameters (e.g. a/b, a/c, coordination number) from the geometry and network models extracted from FIB and TEM images at the nanoscale are assigned into four pore associated phases in PFIB images. An integrated large-scale network can be built following this workflow (Fig. 6).

When the whole sample is considered, the integrated pore network across three scales principally combines two distinct sub-networks. These are a) a global, web-like pore network between phyllosilicate minerals with sizes in the range 6–50 nm and coordination numbers 2–5, and b) a localized, cluster-like connected pore network lying within porous organic matter with sizes in the range 200–800 nm with coordination numbers 5–9 (Fig. 6).

### Pore geometry and network inferred from bulk permeability measurements

**Figure 7:**
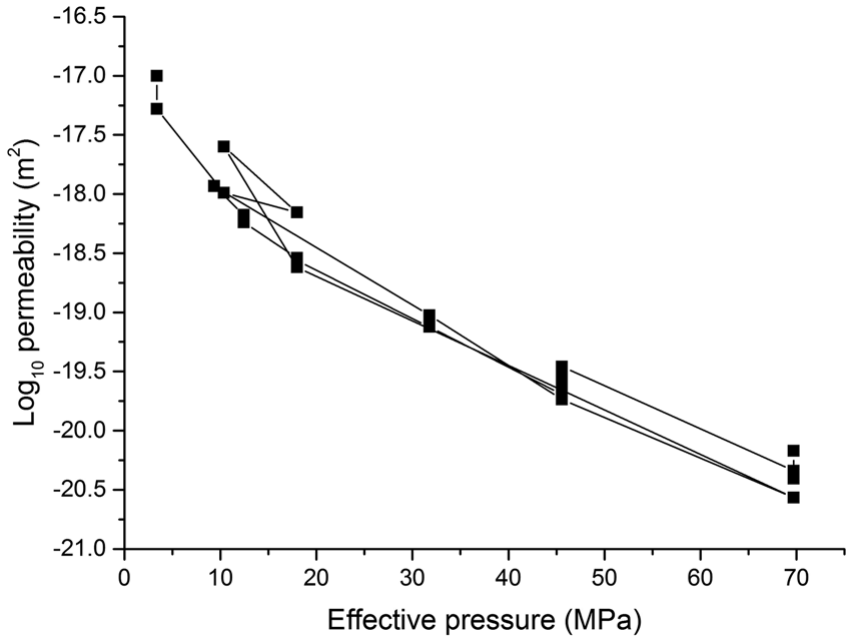
Bedding-parallel permeability to argon gas plotted as a function of effective pressure at a constant pore pressure (23 MPa) in the Darcian flow regime. Measurements made after initial pressure cycling. Three further pressure cycles shown. Standard error in permeability measurements is ±0.2 log units.

We made permeability measurements for this rock using argon gas as permeant for flow parallel to bedding using the oscillating pore pressure technique^30^. Permeabilities range from 1.0 × 10^−17^ to 3.7 × 10^−21^ m^2^ at a constant pore pressure of 23 MPa when the effective pressure is increased from 0 MPa to 70 MPa, demonstrating the low-permeability and high pressure-dependence of this sample (Fig. 7). Permeability normal to bedding is typically about 2 orders of magnitude lower. The high sensitivity of permeability to effective pressure, its low value and its marked anisotropy indicates that transport is dominated by flow through crack-like pores predominantly oriented parallel to bedding.

## Discussion

Pores sizes in shales normally range from a few nanometres to a few microns^28,31^. This study reconstructs from the basic components of the pore network a comprehensive and integrated 3D pore system over the range 2 nm to 3 µm in a Haynesville-Bossier Shale sample, covering the great majority of pores in the selected shale sample. The PCA method used for the geometry and network characterization significantly improved the accuracy of the pore models for the shale system. The imaging method applied combines advanced correlative 3D SEM and STEM imaging techniques, including Xe^+^ plasma FIB, Ga^+^ FIB and STEM tomography, with the field of view ranging from around 30000 µm^3^ (PFIB), 500 µm^3^ (FIB) to around 0.02 µm^3^ (STEM) and corresponding volume-equivalent spatial resolution from around 66 nm, through 39 nm to 2 nm. These EM based techniques, compared with X-ray tomography techniques, provide higher resolution images with improved contrast, and have the potential to be combined with chemical composition mappings^32,33^. In comparison, the best resolution obtainable by X-ray tomography (Nano-CT) is no better than 100 nm (voxel size 50 nm). Also, the optical amplification of Nano-CT limits the contrast obtainable between different phases^34^. Thus it is almost impossible to identify or separate pores from phyllosilicate minerals or organic matter accurately^20^. The combination of the three advanced EM techniques in the present study maximizes the feasibility of imaging and quantification of pore structures in shales.

The present study presents a classification of geometric and network models of porosity according to how the pore spaces are hosted by specific solid phases (Fig. 3) or are formed at their interfaces. The low variability in geometries and coordination numbers of a given pore type makes this approach practical (Fig. 4). The geometry of pores is expected to affect significantly the propagation of induced fractures and porous flow behaviours^10,35,36^, but it has not been possible to evaluate these influences in many other models. The pore geometry and networking demonstrated here has the potential to provide higher accuracy and applicability to petrophysical modelling than an approach based solely on pore-throat sizes.

The implications of pore geometry and network connectivity from this study can be compared with inferences made from experimental permeability measurements made on bulk samples of the same rock as used in this imaging study (Figs 5 and 7). These data and many other permeability measurements on mudstones implies that gas flow and its pressure sensitivity is indeed controlled by pore structures characterized by the fraction of pores with low aspect ratios and aperture widths ranging 20–60 nm^30,37^, which corresponds closely with the imaging results in this study (Fig. 5). Analysis of the permeability results showed that whilst a large portion of the pore volume is hosted within large storage pores that control bulk rock compressibility and account for the greatest fraction of the whole-rock porosity, these have little effect on permeability^38^. The observed high pressure sensitivity of permeability for flow parallel to bedding, coupled with extreme permeability anisotropy, implies that flow takes place via narrow, compressible crack-like pores parallel to bedding that link the large pore volumes that control fluid storage. The low magnitude of observed permeability also requires that the conductive channels be no more than a few nanometres to tens of nanometres in dimension (i.e. the globally connected InterM pores). Flow of gases can occur via a combination of slip flow (at low gas pore pressures, typically <10 MPa) and Darcy viscous flow at higher pore pressures. These results are wholly consistent with the imaging results from the present study, that only the very narrowest and anisotropically shaped (InterM) pores control the transport properties, and link the larger (IntraO and InterOM) pores that provide the fluid storage capacity of the rock.

The research was performed on one shale sample which is considered to be representative of both the sampled formation and more broadly of ‘average’ shale properties. Although this study does not capture the variability in the formation or shales generally, the analytical method can be more widely applied, and linked to sedimentological characterization, for example, compaction, cementation, and the kerogen maturity. A linear relationship between the volumes of these four pore types with the corresponding compositions has been found in Lublin shale samples with similar depositional history^27,39^.

## Conclusions

This study presents a novel combination of 3D SEM and (S)TEM imaging techniques, including Xe^+^ plasma FIB, Ga^+^ FIB and STEM tomography, enabling the quantification of a wide range of pore sizes. This is the first application of Plasma-FIB (PFIB) in shales, and the first study to comprehensively to develop pore geometric and network models in 3D based on experimental characterisation over these critical length scales.

Four pore types (Intra-organic pores, Organic-mineral interface pores, Inter-mineral pores, Intra-mineral pores) were categorized, associated with four solid-phase components, and their geometric shapes characterized. Whilst pores below 10 nm contribute most in number, pores between 10 to 100 nm contribute most to pore network connectivity and hence to fluid flow. Pores between 100 to 2000 nm contribute most in volume and hence to fluid storage capacity. The resultant integrated geometric and network characteristics lead independently to the same conclusions as drawn from experimental permeability measurements made on the same Haynesville-Bossier Shale sample.

The geometry and pore network characterizations lead to an improved understanding of mineral- and organic-matter pore networks in shales covering a wide range of scales. The implications of the results for gas and liquid storage space and transport pathways will be significant in many areas, including shale gas, carbon sequestration, nuclear disposal and possibly geo-thermal energy.

## Materials and Methods

A representative shale sample for typical shale gas reservoirs in North America was used, selected from the Bossier Formation in the Haynesville-Bossier Shale reservoir, currently one of the largest hydrocarbon-producing shale reservoirs in the world. It was characterized as granular minerals (55.3 wt% quartz, calcite, ankerite, albite and pyrite) and phyllosilicate mineral-rich (41.0 wt% illite, chlorite and muscovite.) and carbonate-poor, organic-rich (TOC 3.7 wt%) and gas-mature (Ro 2.3%), determined by X-ray diffraction and proportions quantified by Rietveld analysis.

### Multi-scale 3D image acquisition

Three scales were used: microscale, nanoscale and sub-nanometre scale. In practice these overlap, but we delineate them for the purposes of describing the features we observe and because the scales approximately correspond to the different imaging techniques employed. The corresponding spherical-equivalent voxel diameters, approximately 22 nm, 13 nm and 0.6 nm respectively, were acquired sequentially as the sample size was decreased at each stage of the data collection campaign (Table S1).

The largest scale of 3D images in this study was acquired by Xe^+^ plasma focused ion beam system (PFIB) using FEI Helios PFIB Dual Beam FIB-SEM facility. Then, two sites (organic-rich and mineral-rich) were selected for Ga + focused ion beam system (FIB) imaging, and the image datasets were acquired using a Dual Beam FIB-SEM (Nova NanoLab 600i, FEI, Hillsboro, United States)^40^. Finally, two sites (organic-rich and mineral-rich) were imaged by scanning transmission electron microscope (STEM) tomography, using an FEI Talos TEM operated at a voltage of 200 kV. A high-angle annular dark field (HAADF) tilt series was collected using the Xplore3D acquisition software at angular increments of 1° between ±60°. All the facilities are based at the Electron Microscopy Centre and the Photon Science Institute in School of Materials in University of Manchester.

Accurate pore quantification depends on both the fields of view and the spatial resolutions of 3D images. A single imaging technique combining high spatial resolutions and very large fields of view is not currently available. Therefore, a compromise between these two parameters was required, and the combination of multiple image datasets at multiple scales is one practicable solution (see supplementary materials for more details).

### Image processing and analysis

PFIB and FIB images were aligned, sheared and filtered as a series of block face images before segmentation. TEM tomography images were aligned, reconstructed and filtered as a series of tilted and penetrating sections. A band-pass filter was used to separate out large structures down to 40 pixels (shading correction) and small structures up to 3 pixels (smoothing) by Gaussian filtering in Fourier space. Then, non-local means filters (search window 21, local neighborhood 5, similarity 0.6) and a top-hat filter (kernel 1) were applied for segmentation.

Pores were measured from PFIB images, FIB images and TEM images, as spherical equivalent voxels of diameters 22 nm, 13 nm and 0.6 nm respectively (Table S1). Pores of fewer than 27 voxels (3 × 3 × 3) were ignored in the image analysis owing to the potential for inaccuracies imposed by noise. Therefore, only pores with spherical equivalent diameters >66 nm, >39 nm and >1.8 nm have been considered for evaluation of pore quantification from each volume respectively.

A number of phyllosilicate mineral pores in the TEM tomography volume, which were observed to be touching the image boundaries, could not be imaged completely (Figure S4 in supplementary materials). These truncated phyllosilicate mineral pores were recovered into whole pores based on the thickness measured in TEM tomography and the three axes geometry model deduced from FIB images (Figure S5 in supplementary materials). 3D visualization of the imaging data was conducted using the 3D image processing software Avizo™ (Standard and Fire versions, FEI).

### Pore quantification

Pores, organic matter, granular minerals and phyllosilicate minerals in the 3D images were first segmented into four phases. Subsequently, four types of pore were separated based on the occurrence and relationships of pores and the other framework phases. To identify IntraO and IntraM pores, undefined voxels within these phases are first filled with the ‘Fill-the-holes’ tool. Where pore voxels overlap with organic matter and mineral phase voxels, these are defined as IntraO and IntraM pores, respectively. The same process was performed for IntraM pores by filling voids in the granular mineral particles and overlap with pores. InterO pores were identified where pores had at least one pixel touching the organic matter boundary and one pixel touching a mineral boundary. InterM pores are defined as the remainder of the pores with a boundary touching phyllosilicate minerals, granular minerals or both.

To identify individual pore sizes and connectivity, pores were separated the tools ‘separate objectives’ and ‘pore network model’ were used in the Avizo™ software. These allowed calculation of ‘watersheds’ between pores and hence allowed individual pores to be identified when they formed parts of connected networks.

Pore size distributions were measured by different techniques: PFIB, FIB, TEM, nitrogen adsorption, helium porosity. These techniques cover pore sizes in different ranges. PFIB and FIB measures pores larger than 20 nm; TEM measures pores in the range of 1–30 nm; nitrogen adsorption gives pore sizes between 2–300 nm; helium porosity covers most of pores above 0.14 nm. Although the pore size distribution is not achievable using in helium porosity analysis, it can supplement the volume fractions measured by 3D images and BJH, especially in sizes below 2 nm. The combination of these techniques covers the full range of pore sizes. It is noted that the left end (lowest bin) and right end (highest bin) in sizes of each technique are not reliable due to technique limitations, so reliable ranges were selected in the centre of the range of each technique to construct the overall size distribution (see Fig. 2C).

Geometric and network models are built according to the pore types. Pore geometry was analysed using the principal component analysis (PCA) method see^24^. Compared to the commonly-used geometric elongation method, which quantifies pores according to their longest and shortest dimensions, the PCA method determines dimensions along three orthogonal equivalent ellipsoid axes (Fig. 1). It therefore provides the more specific spatial and orientation information required for a full 3D geometric model.

Principal component analysis (PCA): Pores shapes and orientations were characterized based on the eigenvalues and eigenvectors of the covariance matrix, The axis length ratios of *a/b* and *a/c* were calculated to characterize pore shapes and their orientations relative to the imaging coordinates(*XYZ*, *XY* reference axes are the bedding plane; Fig. 1). Geometric quantifications of pore axial ratios and orientations at three scales were performed separately and then a more specific quantification of four pore types was conducted at nanoscale and sub-nanometre scales using FIB and TEM datasets.

Pore networks are shown as balls and channels (e.g. Fig. 2) in this study, which illustrate pores and throats as spheres and connecting lines respectively. The connectivity of pores along three directions was determined, and the throat lengths and diameters were calculated for the connected network. The coordination number was measured for each pore to evaluate the contribution of individual pores to the network.

### Other laboratory measurements

The measured accessible porosity of the sample used was 7.0% on a 20 mm diameter cylinder with a helium porosimeter. Permeability was measured using argon gas as permeant by the pore pressure oscillation technique^41^ parallel and perpendicular to the bedding on 20 mm diameter samples, oven dried at 60 °C. The effective pressure was stepwise cyclically varied from 0 to 70 MPa at a constant pore pressure of 23 MPa. After the first pressure cycle permeability versus pressure was reproducible, indicating that pressure response was elastic. Pore-size distributions were measured by nitrogen (N_2_) sorption using a surface area analyser (Micromeritics ASAP 2010, Norcross, United States), and the pore size distribution was calculated using the Barrett-Joyner-Halenda (BJH) method^26^.

TOC was measured after rock acidification and organic matter combustion using a carbon analyser at the University of Newcastle (Leco, Michigan, United States). Quantitative X-ray Diffraction (Bruker D8Advance XRD, Billerica, United States) was undertaken in University of Manchester to confirm the mineralogy and identify the phyllosilicate phases present in the samples.

### Data Statement

The datasets generated and analysed during this study are not publicly available due to their large size. They can be made available from the corresponding author on reasonable request.

## Electronic supplementary material

Supplementary materials
