# Supplementary material for: Hierarchical integration of porosity in shales
Source: Sci Rep. 2018 Aug 3;8:11683. doi: 10.1038/s41598-018-30153-x (PMC6076238; doi:10.1038/s41598-018-30153-x)
Supplement: Supplementary file 1 — Supplementary materials [file 41598_2018_30153_MOESM1_ESM.docx]

# Supplementary materials

**Hierarchical integration of porosity in shales**

### Ma, Lin^1,2*^, Slater, Thomas^2^., Dowey, Patrick J. ^1^, Yue, Sheng^3^., Rutter, Ernest. H.,^1^, Taylor, Kevin G.^1^, Lee, Peter D.^3,4*^

^1^ School of Earth and Environmental Sciences, The University of Manchester, Manchester, M13 9PL, UK

^2^ Manchester X-ray Imaging Facility, School of Materials, The University of Manchester, Manchester, M13 9PL, UK

^3^ Research Complex at Harwell, Harwell Campus, Oxfordshire, OX11 0FA, UK

^4^ Department of Mechanical Engineering, University College London, London, WC1E 7JE, UK

*corresponding authors: Lin Ma ([lin.ma@manchester.ac.uk](mailto:lin.ma@manchester.ac.uk)) and Peter D. Lee ([peter.lee@ucl.ac.uk](mailto:peter.lee@ucl.ac.uk) )

### Gallery of examples of four pore types


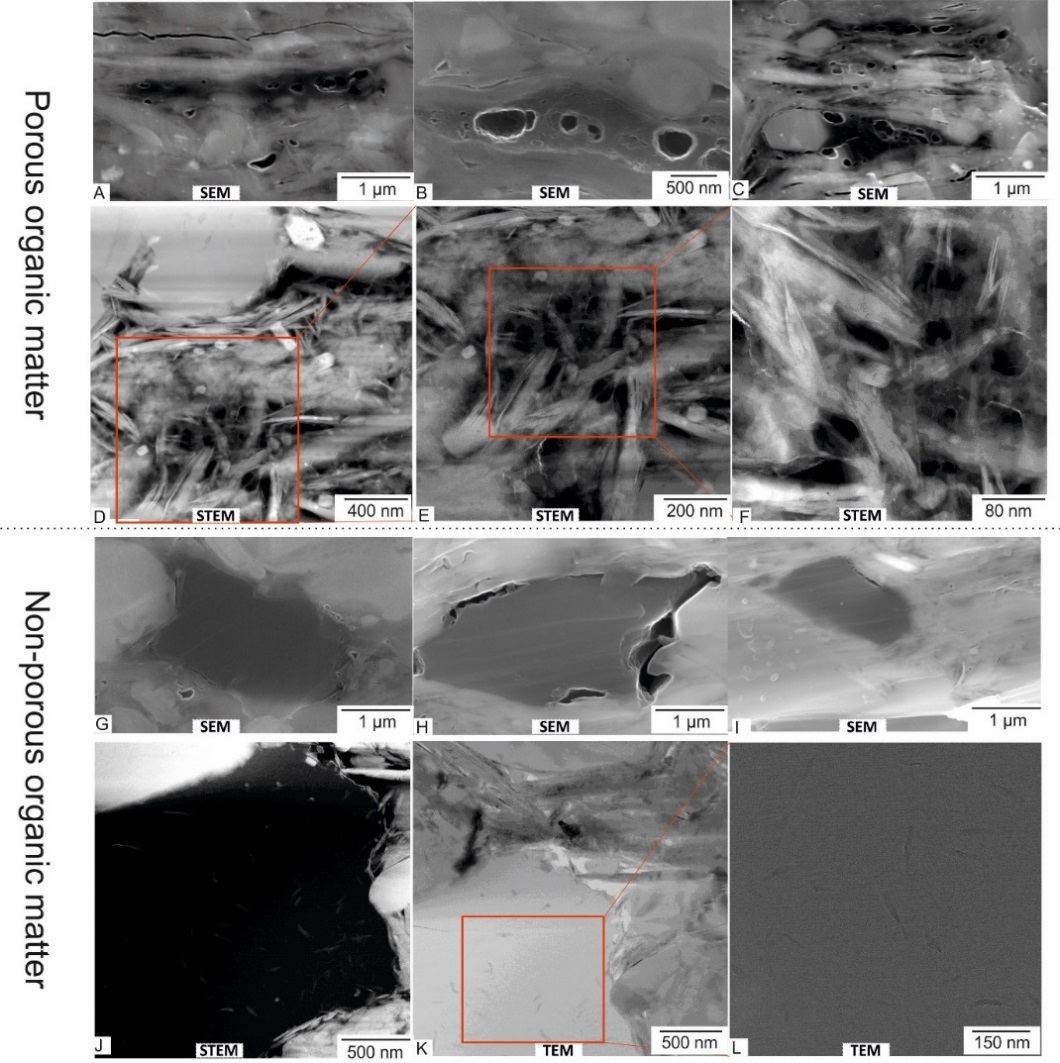


**Figure S1 SEM and (S)TEM images of non-porous organic matter and porous organic matter with varying pore structures.**


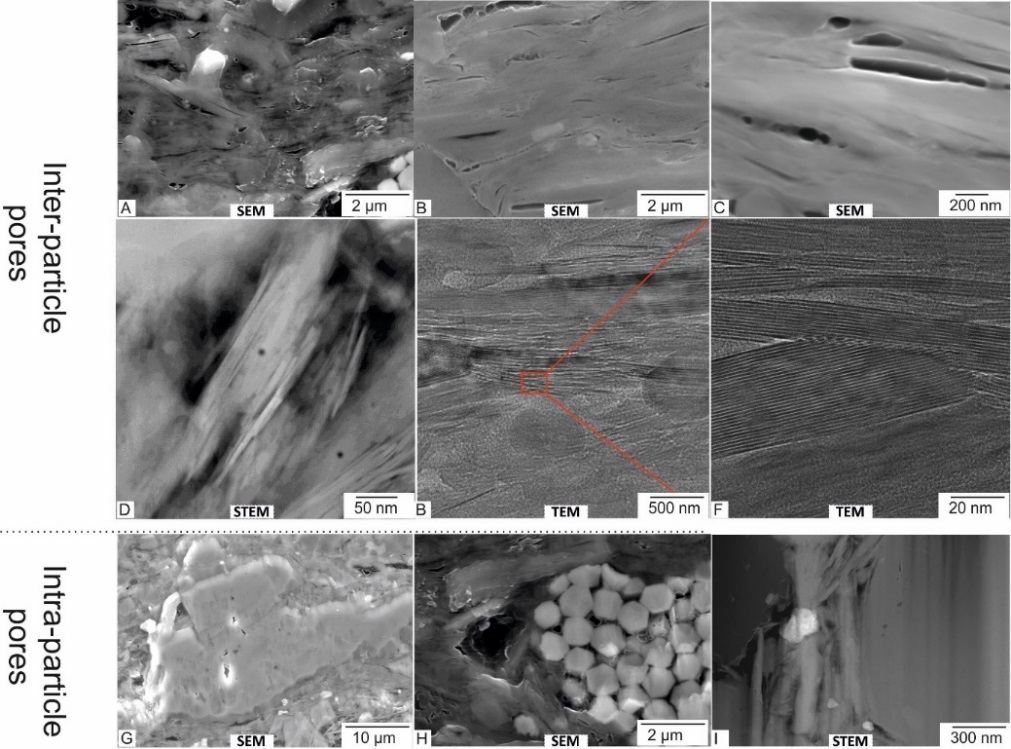


**Figure S2 SEM images and (S)TEM images of phyllosilicate minerals and granular minerals with varying pore structures.**

### Instrumental capabilities and limitations between scales

Image resolution in (S)TEM imaging away from zone-axes is governed primarily by scattering within the sample, which has the effect of broadening of the beam before it reaches the detector. Using established equations of beam broadening and image resolution ([Watanabe, 2011](#_ENREF_40)), a 60 nm thick sample would allow imaging at resolutions no better than approximately 1 nm. When tilting the sample to 60° the thickness of the sample along the electron beam doubles, therefore resulting in a resolution of no better than 3 nm. The resolution in the TEM tomography volume is therefore non-uniform. We estimate the resolution in XY planes to be approximately 1 nm, increasing to above 3 nm in the Z-direction due to increased beam broadening and the effect of the ‘missing wedge’ of information due to limited tilt angles.

Spatial resolution of PFIB and FIB images largely depends on the slice distance on the Z axis. The lack of information between slices in Z direction cannot be avoided for pore analysis. It might lead to a distorted geometry of individual pores, or an interrupted pore network, especially when it only includes a few voxels. The possibility still exists that the two individual pores in the same XY positions in adjacent slices are recognized as one integral pore due to the lack of information between two slices. Orientations of pores below slice distance are not reliable for this reason. Only pores larger than 3 voxels are considered for size quantification and only pores larger than 9 voxels are analyzed for geometry and network model in this study, to reduce the uncertainty of pore analysis in the spatial resolution perspective.

The fields of view in (S)TEM images are limited to the thickness of samples. They are normally less than 200 nm to allow adequate transmission of the electron beam. (S)TEM images in this study are only 60 nm in thickness, so some pores are truncated at the image boundary. These touching boundary pores are removed in geometry analysis (Figure A1B), but are required in network analysis as they bridge between some small pores.

The fields of view of FIB and PFIB depend on the balance of image pixel sizes and number of pixels per 2D slice. FIB images are normally limited to tens of microns with a few nanometre pixel sizes and thousands of pixel numbers in SEM slice. PFIB can provide larger volumes, which are hundreds of microns, but a compromise in pixel sizes should be made. The majority of pores in shales are tens to hundreds of nanometres, so fields of view less than 200 μm are likely to be achieved.

**Table S1 Voxel sizes and corresponding spherical-equivalent diameters for images at three scales**

|  | PFIB | FIB | TEM |
| --- | --- | --- | --- |
| Physical sizes (XYZ µm) | 80 ×65 × 40 | 10 × 10 ×5 | 0.6 × 0.6 × 0.06 |
| Voxel sizes (XYZ nm) | 16× 13 × 50 | 10×10×20 | 0.6 × 0.6 × 0.6 |
| Spherical-equivalent diameter (nm) | 22 | 13 | 0.6 |
| Spherical-equivalent minimum diameter for pore analysis (nm) | 66 | 39 | 1.8 |

### Comparison of PCA methods and geometric elongation approach


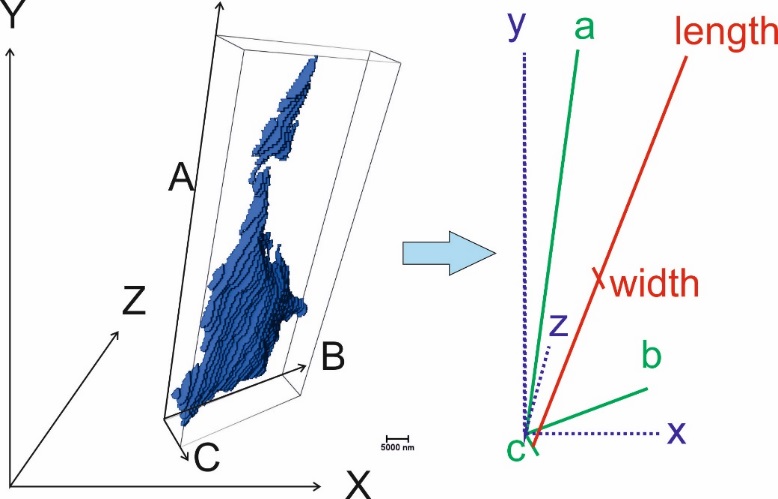


**Figure S3 Schematic diagram (oblique projection) showing the character of the geometry model according to the PCA method (a, b, c), compared to the geometric elongation (length and width) approach. XYZ and ABC are orthogonal coordinate systems. xyz and abc are corresponding length values.**

### Reconstruction of pores touching image boundary


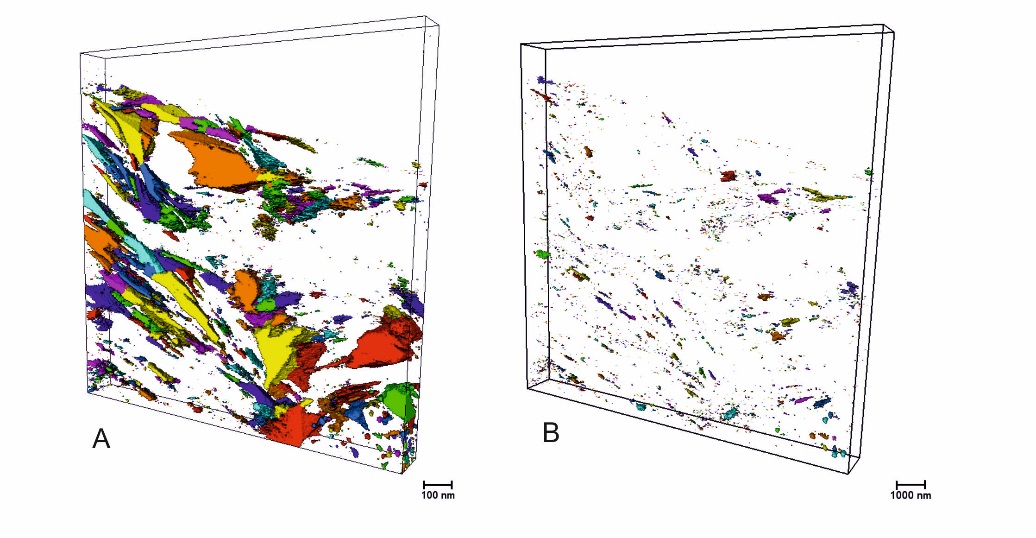


**Figure S4 Reconstruction of pores size distributions from TEM tomography data, A: original pores in TEM, B: non-****touching** **boundary pores.**


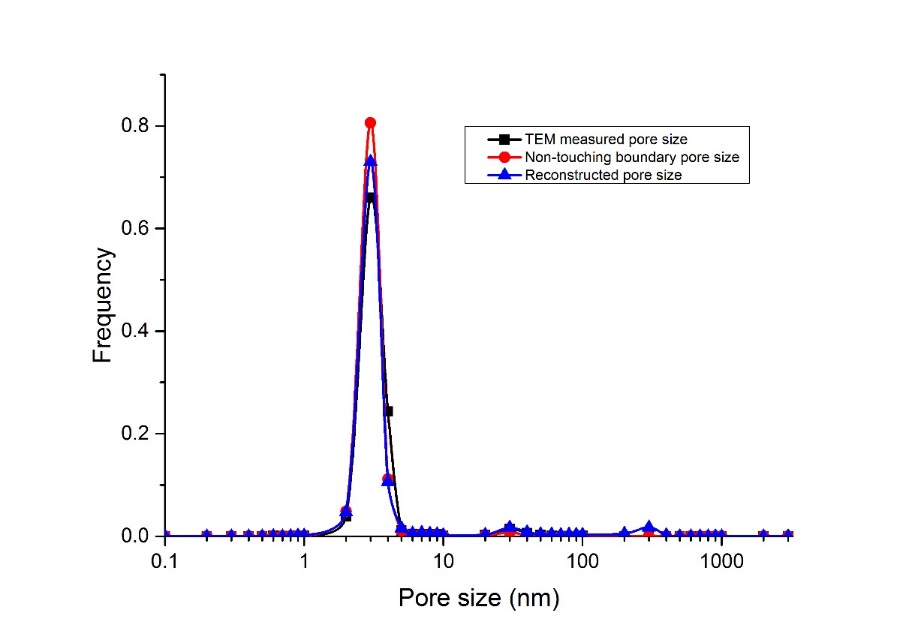


**Figure S5 Comparison of pores size distributions from TEM tomography data, black- pore size distribution directly measured from TEM tomography data, red- non-touching boundary pore size distribution, blue- reconstructed pore size distribution.**
